# Merging Evidence-Based Psychosocial Interventions in Schizophrenia

**DOI:** 10.3390/bs4040437

**Published:** 2014-11-06

**Authors:** Tania Lecomte, Marc Corbière, Stéphanie Simard, Claude Leclerc

**Affiliations:** 1 Department of Psychology, University of Montreal, C-358, 90 Vincent d’Indy Street, C.P. 6128, Succ Centre-Ville, Montreal, QC H3C 3J7, Canada; E-Mail: simardstephanie@hotmail.com; 2 Centre for Action in Work Disability Prevention and Rehabilitation, School of Rehabilitation, Université de Sherbrooke, 150 Place Charles Le Moyne, Bureau 200, Longueuil, QC J4K 0A8, Canada; E-Mail: marc.corbiere@usherbrooke.ca; 3 Université du Québec à Trois-Rivières, 3351 Boul Des Forges, Trois-Rivières, QC G9A 5H7, Canada; E-Mail: claude.leclerc@uqtr.ca

**Keywords:** evidence-based intervention, schizophrenia, CBT, skills training, family psychoeduaction, supported employment, cognitive remediation

## Abstract

Psychosocial interventions are an essential part of the treatment for people with severe mental illness such as schizophrenia. The criteria regarding what makes an intervention “evidence-based” along with a current list of evidence-based interventions are presented. Although many evidence-based interventions exist, implementation studies reveal that few, if any, are ever implemented in a given setting. Various theories and approaches have been developed to better understand and overcome implementation obstacles. Among these, merging two evidence-based interventions, or offering an evidence-based intervention within an evidence-based service, are increasingly being reported and studied in the literature. Five such merges are presented, along with their empirical support: cognitive behavior therapy (CBT) with skills training; CBT and family psychoeducation; supported employment (SE) and skills training; SE and cognitive remediation; and SE and CBT.

## 1. Introduction

Psychosocial interventions are an essential part of the treatment and recovery of people with severe mental illness such as schizophrenia. It is well-accepted that medication alone is not sufficient to help with the recovery and various issues a person might encounter when attempting to lead a satisfying life in the community. Some practices have gathered sufficient empirical data to be strongly recommended by national guidelines such as the PORT (Patient outcome research team) [1], or the NICE (TheNational Institute for Health and Care Excellence) [2] guidelines in the UK, whereas others are considered promising until more studies support its effect. In order for a psychosocial practice, namely an intervention or program (an intervention is specific treatment with a targeted therapeutic aim whereas a program typically includes various interventions), to be considered evidence-based, it must not only have accumulated sufficient empirical proof of its efficacy (often in the context of at least two randomized controlled trials) but it must answer a need (such as improve functioning, overcome a deficit or help to cope with symptoms) and be standardized in order to be easily replicable [3]. The 2009 PORT report on psychosocial practices for schizophrenia highlighted eight interventions or programs that could be considered evidence-based [1]. These were in terms of interventions: family psychoeducation, cognitive behaviour therapy, social skills training, weight management, and token economy. In terms of programs, they described: intensive community treatment, supported employment and integrated dual-disorder programs (with first episode programs almost meeting criteria). Since, Mueser *et al.* [3] has added cognitive remediation, which has demonstrated in various meta-analyses its efficacy in helping individuals overcome cognitive deficits in areas such as memory, attention or problem solving. Although all of the above-mentioned interventions and programs are considered evidence-based and can truly improve the lives of people with severe mental illnesses, implementation studies reveal that few, if any, are ever implemented in a given setting [4]. For some interventions, this is understandable—for instance token economy is only effective in changing problematic behaviors when used in a closed and controlled environment, like a forensic ward [5]. However, most of the other interventions and programs have demonstrated positive effects in improving symptoms, deficits, and community functioning and are under-utilized. A recent review on implementation of evidence-based psychosocial interventions and programs in psychiatry describes various strategies that have been used in order to improve implementation of one or many evidence-based practice [4]. Of the most common strategies, large demonstration projects and effectiveness trials run by researchers, broad service reforms based on new government policies and national agency-led initiatives were identified. Few of these implementation strategies have been studied over the long-term, and fewer even have considered consumer outcomes [4]. Other smaller-scale implementation strategies have also been developed, when large-scale funding was not available. For instance, supported employment programs’ implementation have been facilitated by a group of researchers and clinicians [6] offering standardized training, consultation and encouraging the use of their implementation fidelity scales (The *Quality of Supported Employment Implementation Scale* (QSEIS) [7] or the *Individual Placement and Support* (IPS) fidelity scale [8]. In the UK, US and Canada, the implementation of CBT for psychosis has been improved by offering the clinical training to mental health professionals from various backgrounds (e.g., nurses, occupational therapists), offering structured manuals, and brief training sessions [9,10] rather than limiting the treatment delivery to clinical psychologists.

Another approach to improving implementation of evidence-based psychosocial interventions or programs that is being seen more and more in the literature, particularly in the last decade, is to merge two evidence-based interventions, or to offer an evidence-based intervention within an evidence-based program. Are considered merges both: (a) the simultaneous delivery of two practices (two interventions or an intervention and a program) and, (b) modified practices: interventions changed in order become integrated with another intervention or within a program. In settings with limited means, offering such merges has the advantage of targeting more than one therapeutic goal at once (e.g., cognitive distortion and work integration). Some of the merges have been developed in order to adapt an evidenced-based intervention to a specific population group or setting (such as older consumers). However, most of the merges have been developed with the hope of improving the effectiveness of an evidenced-based program by adding an empirically recognized intervention. It is important to mention that few of the merges presented here simply “co-deliver” interventions—most are well-integrated merges or at least aim at truly integrating the interventions and programs together. The following are descriptions of such merges.

## 2. Cognitive Behavior Therapy (CBT) and Social Skills Training

Granholm and colleagues [11,12] have merged two evidence-based psychosocial interventions to cater to the specific needs of aging individuals with psychosis who might be struggling not only with symptoms but also with social and cognitive deficits. CBT for psychosis has been studied in over 40 randomized controlled trials and various meta-analyses since the first promising studies of the 1990s [13]. Overall, most studies have found that CBT for psychosis is effective in reducing symptoms and improving other indexes of well-being, often with stronger results compared to other interventions overtime than at post-treatment [14,15,16]. However, as in most psychotherapy studies, the most rigorous studies often reveal smaller effect sizes than the non-controlled studies [17]. CBT for psychosis aims at modifying dysfunctional beliefs by helping the person understand the link between perceptions, beliefs and emotional and behavioural reactions. CBT also helps the person question the evidence supporting his beliefs (whether they are psychotic or not). Furthermore, CBT brings the person to self-observe himself, his thoughts and behaviors, and explores various coping strategies the person might use when dealing with distressful thoughts or voices. Finally, CBT for psychosis takes into account cognitive biases a person might have and aims at modifying those biases, by seeking alternatives instead of jumping to conclusions, for instance.

CBT for psychosis has been adapted for various clienteles, used with individuals at high risk of developing psychosis, individuals with early psychosis as well as older individuals with a long history of schizophrenia. This latter group was of particular interest for Granholm and colleagues [12] who wished to offer CBT for psychosis in a format that would be appealing and adapted to clients who were often isolated, and who might have difficulties grasping some CBT concepts, given their cognitive deficits. They therefore decided to include social skills training to the CBT treatment and offer the merged treatment in a group format.

Social skills training has been around since the 1980s in the USA and was considered especially useful for helping people reintegrate society after a long period of institutionalization. The goal behind skills training is to offer skills that are deemed essential to interact with others, manage one’s medication and symptoms, as well as problem solve in different contexts. Skills training is based on Bandura’s self-efficacy theory [18] and uses repetition and positive reinforcement to help people acquire and remember new skills. To date, over 23 randomized controlled trials have shown that skills training can help acquire skills, decrease negative symptoms, and has a moderate impact on independent living skills [19,20]. Skills training can be offered individually but works best in groups, with the use of multiple role-plays preparing for real-life interactions.

### Merging CBT and Skills Training

Granholm and colleagues [11,12] developed a group CBT/skills training approach that focuses on CBT for psychosis techniques, such as checking for facts, but presents these in a skills training manner (*i.e.*, a lot of repetition, wallet cards with key words/concepts, use of a big flag in the group to “flag” the beliefs without apparent facts or proof). The group included modules that were repeated over time, enabling the participants to go over the content more than once and allowing new participants to enter the group at any given moment. Although the results did not show an improvement in positive symptoms, it did show improvements in functioning and negative symptoms [11,12]. Of importance, the participants were able to remember the concepts and CBT techniques regardless of the severity of their cognitive deficits.

## 3. CBT and Family Psychoeducation

Leclerc and Lecomte [21] have recently published promising preliminary data on 40 family members who received a merged group CBT/psychoeducation family intervention. Family intervention, in particular family psychoeducation, is recognized as one of the evidence-based interventions with the most empirical support, especially regarding decreasing rehospitalization rates [3]. More than 50 randomized controlled trials have been published to date supporting the effects of family psychoeducation on increased medication adherence, and decreased stress and symptoms in those receiving psychiatric care [15,22]. As for their family members, these same studies report decreased perceived burden and psychological distress. Most family interventions last an average of six to nine months and offer: information on symptoms and mental illness, recommendations for dealing with crises, emotional support, and coping skills to deal with symptoms and mental illness [23]. Family interventions can be offered to each individual family or multiple families together, with or without the family member receiving psychiatric care. Many family intervention manuals were developed in the 1990s and do not address recent concepts such as recovery and tend to focus mostly on medication, chronicity, and symptoms. During a recent trial on CBT for early psychosis, many family members asked to learn more about CBT for psychosis and how they could use the tools in their lives. We therefore developed the family psychoeducation/CBT module entitled WITH (Wellness-Inform-Talk-Help) [21]. The module can be offered in parallel to the CBT for psychosis groups, *i.e.*, during 24, hourly multiple family sessions, or can be offered in a more intensive format: eight two-hour multiple family sessions (covering 16 activities in the module). Each multiple family group typically consists of an average of 10 parents and two co-therapists. The content of the group is psychoeducational in that it addresses concepts such as recovery, expressed emotions, parental role, personal limits, and expectations, but it is also considered CBT given that the participants learn about CBT principles and techniques and apply them to their own lives during the group and at home (homework). The parents therefore learn to not only use the skills learned with their family member with a mental illness, but also use them with themselves when they are experiencing distress for instance.

### Merging CBT and Family Psychoeducation

The intensive (eight two-hour sessions) format was recently studied in a non-controlled study [21] whereby the 40 parents who participated showed significant clinical improvements in psychological distress, namely in psychoticism, depression and interpersonal sensitivity compared to their baseline scores. Qualitative data obtained revealed that parents appreciated the group, found it helpful, and they had integrated recovery as well as CBT notions and skills in order to improve their relationship with their family member receiving psychiatric services. The group format was especially appreciated, as well as the information covered in the module. Although more studies are warranted in order to compare the WITH multiple family intervention to other family interventions, social workers offering the group anecdotally mentioned that their previous multiple family psychoeducational intervention had a retention rate of only 20% of participants from the first to the last session whereas WITH had a retention rate of 80%. The merged intervention has the advantage of covering essential elements of family psychoeducation for psychosis, including updated information on recovery, and also offers concrete CBT tools that can be useful for the person with a mental illness as well for their family members.

## 4. Supported Employment and Other Evidence-Based Interventions

Supported employment is another evidence-based program that has attracted a few merges over the past decade. Supported employment programs help people with severe mental illness obtain real-world competitive employment, with regular wages, based on their clients’ preferences [6]. Employment specialists working in supported employment programs aim at quickly finding regular paid work for their clients, and offer them unlimited support according to their needs at work. Supported employment programs are recognized as being evidence-based with more than 15 trials in various countries having demonstrated that SE programs are more efficacious in helping people with severe mental illness obtain regular jobs than other vocational or rehabilitation programs [6]. Nonetheless, there is room for improvement given that on average, in North America, between 40%–60% of the clients in SE programs obtain regular jobs and most jobs are only kept for three to five months. Many reasons have been suggested to explain why some individuals might struggle in finding work or in maintaining their jobs. Some have suggested lack of appropriate work-related social skills, others that cognitive deficits impede on work performance, and others still that people with mental health problems might hold irrational beliefs about themselves and the workplace.

### Merging Social Skills Training and Supported Employment Programs

The first merge proposed was to offer social skills training that was work specific to people registered in a supported employment program. Charles Wallace [24] developed the *Workplace Fundamentals*, aiming at helping participants recognize the advantages of work in their lives, their potential stressors at work, how to problem solve various work-related situations, and how to avoid drugs and alcohol to maintain their jobs. The module is offered over the course of 24 sessions, typically twice a week, in groups of six to eight participants. As with most social skills training, the goal is to develop spontaneous behaviors and therefore involves multiple role-plays and repetitive behaviors. Two studies were conducted to verify its efficacy in improving job tenure. The first, including 34 participants, showed improved job tenure and better work satisfaction for those having received the skills training + SE program compared to SE program alone [24], whereas the second study did not show any differences between the two conditions on work outcomes (but reported that the sample was not typical of most studies with higher education and longer tenure, with rates close to one year for their first job) [25]. The participants receiving both conditions did show greater knowledge regarding their work setting, stressors and showed better problem solving abilities than those receiving only the SE program.

## 5. Cognitive Remediation and Supported Employment

Another explanation for poor work tenure in people receiving SE programs pertains to cognitive deficits. Cognitive deficits are well documented in people with severe mental illness, namely regarding deficits in memory, attention, speed of processing and various executive functioning tasks, and can make performing at work difficult. Various cognitive remediation programs and strategies have been developed over the years with more than 40 randomized controlled trials supporting its efficacy in improving cognitive skills and overall functioning [26]. Cognitive remediation can take many forms: paper-pencil tasks, computer tasks, group training, or training in real-world tasks (using errorless learning, for instance [27]). Although some cognitive remediation can include modifying the environment to compensate for the person’s most important cognitive deficit, most cognitive remediation programs aim at improving cognitive deficits to the point that they no longer interfere with work performance.

### Merging Cognitive Remediation and Supported Employment Programs

McGurk and colleagues developed a cognitive remediation program called *Thinking Skills for Work* specifically for people registered in supported employment programs [28]. The program involves individual computerized training (using CogPack) for an average of 24 hours over the course of 12 weeks, along with cognitively-informed job support consultation with the employment specialist. The computerized program aims at improving attention, concentration, psychomotor speed, learning and memory as well as executive functions. The consultation aims at targeting jobs or at modifying the work environment as needed according to the person’s performance and progress during the cognitive remediation training. Results at the two to three year post- cognitive remediation follow-ups revealed that those who had received the cognitive remediation program had improved on the cognitive tasks and had superior job retention rates than the control condition (registered in supported employment programs only) [29]. These results were however not found for those who presented with comorbid substance use disorders—their work outcomes were poor regardless of the extra treatment added [30].

## 6. CBT and Supported Employment

A potential obstacle to job maintenance in people registered in supported employment is dysfunctional beliefs regarding the workplace and one’s own abilities. Individuals with severe mental illness who have been away from the job market for some time can hold beliefs and act in ways that are deleterious for their work integration, and could be influenced by lack of confidence, jumping to conclusions bias, and poor coping skills, to name a few. As mentioned previously, CBT has proven efficacious in modifying beliefs and cognitive biases and helps in developing better coping strategies when dealing with stressful situations. CBT has also been modified by Davis and colleagues [31] to target work beliefs and behaviours in a transitional vocational program for veterans with severe mental illness. This program, entitled IVIP, has demonstrated improvements in work performance and job maintenance in those receiving the group IVIP compared to those participating in the vocational program alone [32]. These results were also replicated in a larger trial [33].

### Merging CBT and Supported Employment Programs

Lecomte, Corbière, Titone and Lysaker [34] developed a brief CBT group intervention, inspired by the IVIP, but specifically tailored for people in supported employment programs called CBT-SE. The CBT-SE intervention is offered during 8 sessions over the course of one month, in order to ensure that the group does not impede on the rapid job search principle of supported employment programs. The content covered many essential aspects linked to the workplace, such as recognizing and managing one’s stressors at work, determining and modifying dysfunctional thoughts (e.g., not jumping to conclusions, finding alternatives, seeking facts), overcoming obstacles (e.g., problem solving), improving one’s self-esteem as a worker recognizing strengths and qualities), dealing with criticism, using positive assertiveness, finding coping strategies (for symptoms and stress) to use at work, negotiating work accommodations and overcoming stigma. Although the results from the trial of 160 participants are not yet available, preliminary data have been published on 24 participants [35] and suggest that the CBT-SE intervention is feasible, and acceptable, with good attendance and positive feedback regarding the group’s usefulness. In terms of work outcomes, 50% of all participants in both conditions found competitive work but those in the CBT-SE condition were more likely to work more hours per week and for more consecutive weeks than those in the supported employment program alone. These preliminary results are promising, although results from the full trial are needed before concluding that CBT-SE is efficacious in improving job tenure.

## 7. Conclusions

Evidence-based psychosocial practices for individuals with severe mental illness can greatly improve people’s lives but are unfortunately scarcely implemented. When large-scale governmental or agency supported implementation initiatives are not available, clinical or community settings who are tempted to offer one evidence-based program or intervention could also opt for a merged intervention. Merged interventions have the advantage of targeting two sets of skills at once, and could therefore generalize in other aspects of the person’s life. For instance, individuals having received cognitive remediation within a supported employment program [28] could see improvements in other areas of their lives, outside of work, from their improved memory and attention skills. Similarly, the cognitive behavioural strategies used in the CBT-SE skills [34] are similar to those used in more general CBT for psychosis treatments and could be used to help the person assess situations differently at work as well as outside of work, with friends or family for instance.

This article aimed at presenting some merges of evidence-based programs but is in no way exhaustive. Other merges exist, such as social skills training with token economy for substance misuse [36] cognitive remediation with social skills training (e.g., Integrated Psychological Therapy - IPT [37]) or social cognitive training with CBT and skills training (*i.e.*, Social Cognition and Interaction Training—SCIT [38]). These programs are however described as distinct and unique programs, not as merges of evidence-based interventions. Although evidence-based interventions are empirically supported, their impact on various outcomes can likely be improved by adding elements from other evidence-based interventions, or by offering them within an evidence-based program, as was demonstrated here. Although some of the proposed merged interventions have only been studied in small or uncontrolled studies so far, the strong empirical support for the non-merged evidence-based interventions from which they are derived and the preliminary data available so far is quite encouraging. Future studies on merged evidence-based interventions are warranted, particularly in terms of trials assessing the effectiveness of offering such interventions simultaneously rather than separately and at different times. Furthermore, studies should also consider measuring the level of integration of the practices in order to determine if closely-knit merges are more effective than less integrated practices. Finally, studies should also investigate if these merges increase or not generalization of the skills to other life domains.
